# Enhanced light extraction efficiency and viewing angle characteristics of microcavity OLEDs by using a diffusion layer

**DOI:** 10.1038/s41598-021-82753-9

**Published:** 2021-02-09

**Authors:** Cheol Hwee Park, Shin Woo Kang, Sun-Gyu Jung, Dong Jun Lee, Young Wook Park, Byeong-Kwon Ju

**Affiliations:** 1 Display and Nanosystem Laboratory, Department of Electrical Engineering, Korea University, 145, Anam-ro, Seongbuk-gu, Seoul, 02841 Republic of Korea; 2 Nano and Organic-Electronics Laboratory, Department of Display and Semiconductor Engineering, Sun Moon University, Asan, Chungcheongnam-do 31460 Republic of Korea

**Keywords:** Electrical and electronic engineering, Organic LEDs, Nanocavities, Photonic devices

## Abstract

The viewing angle characteristics and light extraction efficiency of organic light-emitting diodes (OLEDs) with a micro-cavity structure were enhanced. This was accomplished by inserting a diffusion layer composed of nano-sized structures of a transparent polymer poly(methyl methacrylate) (PMMA) combined with a zinc oxide (ZnO) semi-planarization layer with a high refractive index (n = 2.1) into the devices. The PMMA nanostructures were fabricated by employing a reactive ion etching (RIE) process. The height and density of the PMMA nanostructures were controlled by varying the speed at which the PMMA was spin-coated onto the substrate. The insertion of the diffusion layer into the micro-cavity OLEDs (MC-OLEDs) improved the external quantum efficiency (EQE) by as much as 17% when compared to that of a MC-OLED without a diffusion layer. Furthermore, adjustment of the viewing angle from 0° to 60° halved the peak shift distance of the electroluminescence (EL) spectra from 42 to 20 nm. Additionally, changing the viewing angle from 0° to 60° changed the color coordinate movement distance of the MC-OLED with the diffusion layer to 0.078, less than half of the distance of the MC-OLED without the diffusion layer (0.165).

## Introduction

Organic light-emitting diodes (OLEDs) are being extensively examined as next-generation technologies in the field of display and solid-state lighting owing to their low power consumption, high color purity and gamut, fast response, and applicability to flexible display devices^1–5^. However, only approximately 20% of the light generated by OLEDs can be extracted. Light loss is mainly due to total internal reflection (TIR) in the multilayer stack of an OLED with deferent refractive indices and is attributed to the surface plasmon polariton (SPP) mode at the organic/metal reflective electrode interface. Thus, approximately 80% of the light is trapped in the glass substrate as the substrate mode, the indium tin oxide (ITO) anode and organic layers as the waveguide mode, and the organic/metal electrode interface as the SPP mode^6–8^. Significant efforts have therefore been devoted to extracting more light from within the interior of OLEDs. The light trapped in the substrate mode that results from the TIR at the interface between the glass substrate (n = 1.5) and air (n = 1.0) was extracted by using an external scattering layer, patterned glass, an array of microlenses, or a textured mesh surface^9–16^. Furthermore, the light trapped in the waveguide mode, which results from the TIR at the interface between the ITO (n = 1.9) and glass substrate (n = 1.5), was extracted by utilizing a randomly distributed nano-sized structure, an internal scattering layer, a periodic photonic crystal, or a layer with a low refractive index^17–23^. However, to a certain extent, the aforementioned external and internal light extraction technologies are problematic in that they adversely affect the display and are dependent on certain wavelengths and viewing angles, experience the pixel blurring effect, and require an expensive and complicated fabrication process to achieve the desired light extraction^24,25^. Thus, most commercial products based on OLEDs use a micro-cavity structure to enhance the efficiency of the devices because a micro-cavity structure is simple to fabricate and functionally effective^26,27^. However, a micro-cavity structure has a critical disadvantage in that the emission pattern becomes narrower than the Lambertian distribution because of the Purcell effect. Furthermore, the color coordinates and the peaks of the electroluminescence (EL) spectra undergo shift depending on the angle at which the display is viewed^28^. Clearly, it is necessary to improve the characteristics of the micro-cavity based on the viewing angle without affecting the improvement in the light efficiency resulting from the cavity effect.

In this study, we used a simple reactive ion etching (RIE) process to fabricate a transparent layer consisting of nano-sized structures of a polymeric material. We subsequently inserted this layer between the glass substrate and the indium zinc oxide (IZO) anode of the micro-cavity OLED (MC-OLED) device in conjunction with a semi-planarization layer to form a diffusion layer with a high refractive index to lessen the viewing angle dependency of the MC-OLED. The semi-planarization layer additionally serves to enhance the optical efficiency of the MC-OLED compared with that of a conventional MC-OLED device.

## Results and discussion

### Optimization of a diffusion layer with a nano-sized structure

**Figure 1:**
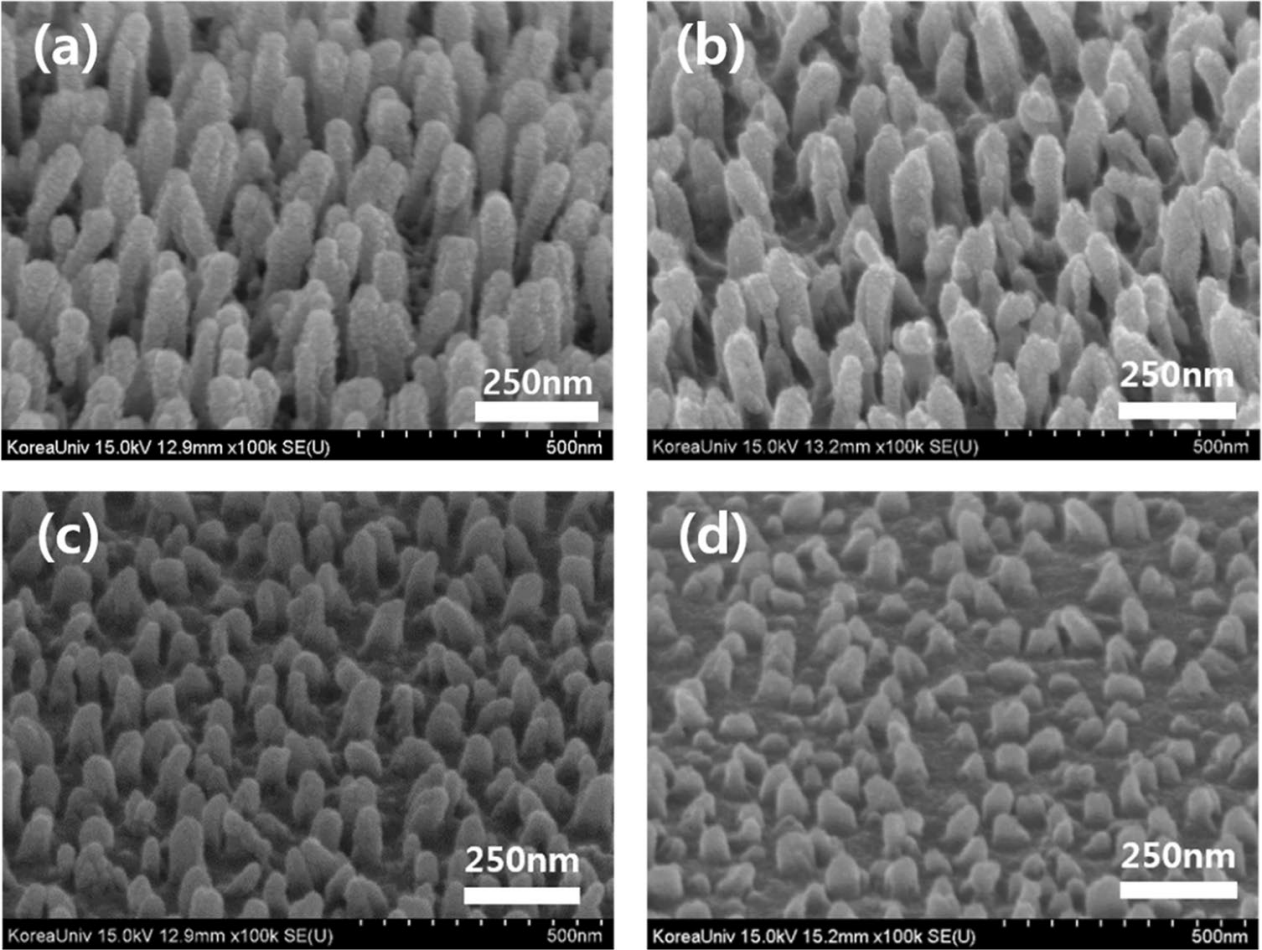
FE-SEM images of the fabricated nano-sized structures of the PMMA layer by varying the speed of the spin coater at (**a**) 1500, (**b**) 1750, (**c**) 2000, and (**d**) 2500 rpm.

**Figure 2:**
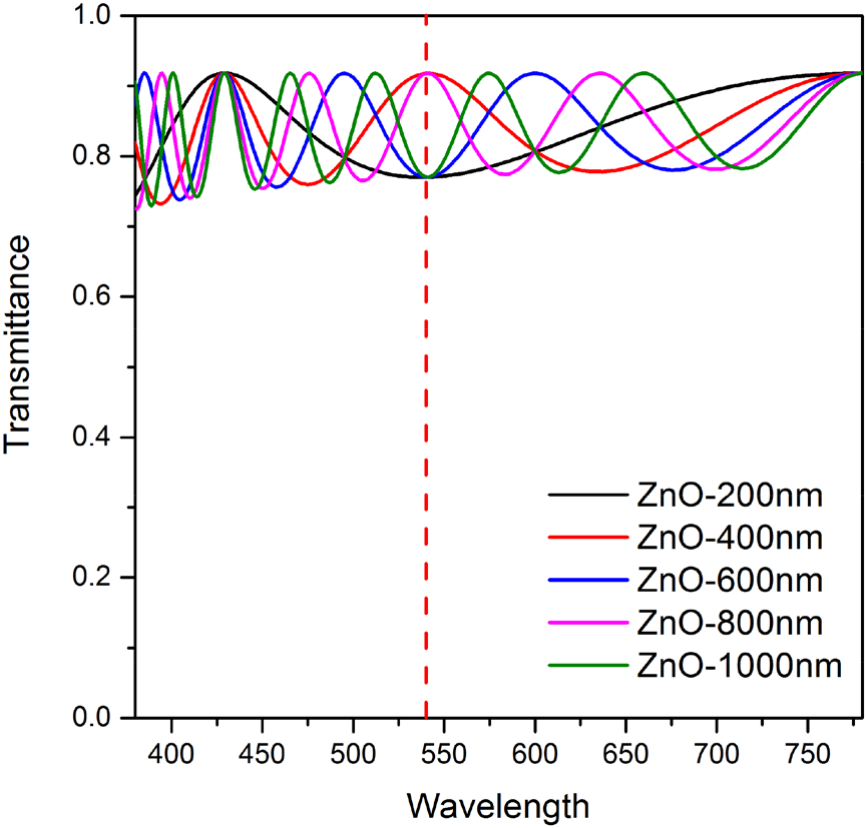
Simulated transmittance by varying the thickness of the ZnO layer.

Figure 1 shows the FE-SEM images of the fabricated nano-sized PMMA structures that were obtained by varying the spin coating speed, which enables the height and density of these structures to be controlled. The FE-SEM images verified the height of these nano-sized structures to be in the range 120–280 nm, and the images confirm that an increase in the spin-coating speed decreases the height and density of the nano-sized structures. Furthermore, the nano-sized structures are randomly orientated, thereby presumably causing significant scattering at the wavelengths of visible light without critical diffraction in a certain direction such as that in periodic photonic crystals^22^. Table 1 summarizes the approximate height of the nano-sized structures that were obtained by depositing a layer of PMMA on the substrate by spin coating at 1500–2500 rpm. However, direct deposition of the IZO anode on this nano-sized structure may result in an electrical short circuit because of the surface roughness of the structure. This would ultimately have a negative impact upon the electrical stability and yield of the OLED device. Therefore, it is necessary to additionally deposit a (semi-)planarization layer on the nano-sized structure to reduce its surface roughness such that the structure is appropriate for incorporation into an OLED device. Materials that would constitute a suitable planarization layer between the nano-sized structures and the IZO anode would preferably require a refractive index that exceeds that of the IZO (n = 2.0)^29^ to avoid an additional waveguide mode between the IZO anode and the interface with the planarization layer. Thus, we selected zinc oxide (ZnO), noted for its high refractive index (n = 2.1), as the material for the semi-planarization layer and examined the transmittance of the ZnO layer by using optical simulation to optimize the thickness of the semi-planarization layer. The simulation was designed by flat structure, and that was only to check the effect of thin-film interface by the ZnO thickness. The results are shown in Fig. 2, which shows that, when the thickness of the ZnO layer changes, the transmittance based on the wavelength changes to wavy owing to the interference effect of thin-film interface between the ZnO layer and IZO layer. In the study, we used green OLEDs with an emission wavelength of 540 nm. Examination of the transmittance at this wavelength revealed that the highest transmittance (approximately 90%) is obtained for layer thicknesses of 400 and 800 nm. Based on the results of our simulation, we finally deposited a 400-nm-thick ZnO semi-planarization layer on the nano-sized structures by using radio frequency (RF) sputtering.

**Table 1:** Height and density of the nano-sized structures of PMMA as a function of the spin coating speed.

Spin coating rpm	(a) 1500	(b) 1750	(c) 2000	(d) 2500
Approximate height of structure	280 nm	250 nm	180 nm	120 nm
Density of nano-sized structure	132 ea/μm^2^	120 ea/μm^2^	108 ea/μm^2^	100 ea/μm^2^

**Figure 3:**
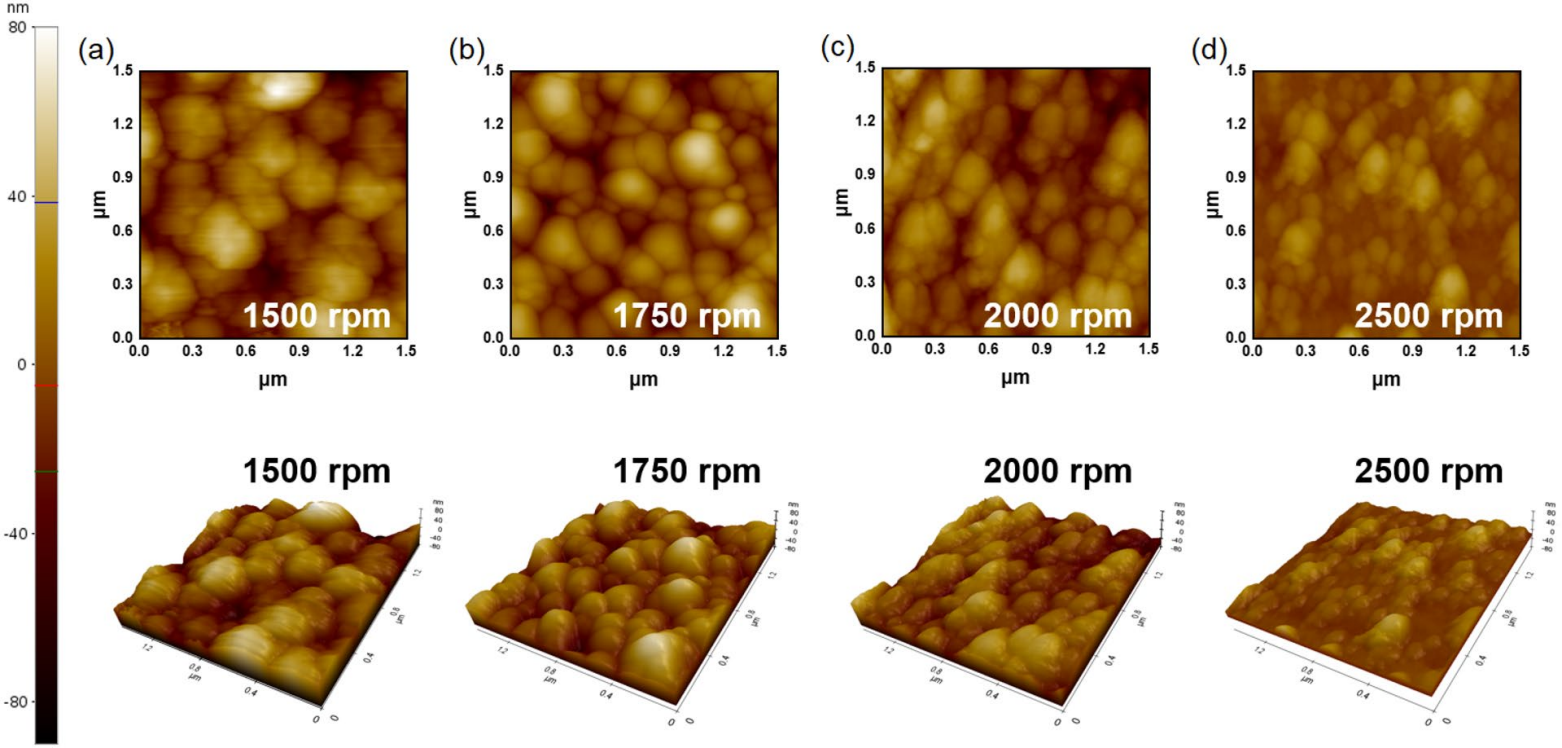
AFM image of ZnO according to PMMA spin coating speed (**a**) 1500, (**b**) 1750, (**c**) 2000, and (**d**) 2500 rpm.

The morphological properties of the deposited ZnO semi-planarization layer on the PMMA layer were analyzed using an atomic force microscope (AFM). Figure 3 shows AFM images of deposited ZnO semi-planarization layer on PMMA spin-coated from 1500 to 2500 rpm, respectively. Table 2 summarizes the Rpv and Rq of the ZnO semi-planarization layer for each rpm measured by AFM. By comparing the Rpv in Table 2, and the SEM results of the PMMA nanostructure layer in Table 1, the height of the nanostructure is decreased on average 48.5%. As confirmed in the FE-SEM image, the increase in the PMMA spin-coating speed decreased the nanostructure height. Likewise, Rpv and Rq decreased as the spin coating speed increased in the AFM results with the ZnO semi-planarization layer on PMMA.

**Table 2:** Summarize of the AFM results of ZnO according to PMMA spin coating speed 1500–2500 rpm.

Spin coating rpm	(a) 1500	(b) 1750	(c) 2000	(d) 2500
Rpv	163 nm	126 nm	114 nm	64 nm
Rq	22.78 nm	19.54 nm	18.64 nm	10.46 nm

Subsequently, we inserted a diffusion layer (deposited under four different conditions) into conventional OLEDs with the aim of evaluating its characteristics to confirm the scattering effect of the diffusion layer (nano-sized structures + semi-planarization layer). The results of the evaluation of the EL characteristics of OLEDs with this diffusion layer indicated that deposition of the layer by spin coating at 1750 and 2000 rpm served to optimize the optical efficiency. (Details of the experiment are presented in Supplementary Fig. S1 and Supplementary Table S1 online). Therefore, a diffusion layer deposited at 1750 and 2000 rpm was subsequently used to improve the viewing angle characteristics of MC-OLEDs.

### Structure of MC-OLEDs

The thickness of the organic structure in the MC-OLEDs ranges from several tens to hundreds of nanometers (termed the cavity length) and the structure is positioned between two double-sided reflective electrodes. In the structure, light resonates between the two reflective electrodes, and the light of a specific wavelength that satisfies the resonance condition of constructive interference is extracted with a strong intensity based on the cavity length. Therefore, it is extremely important to determine the cavity length to obtain intense light of the desired wavelength from OLEDs with a micro-cavity structure. Briefly, the construction conditions of the MC-OLEDs are derived from Fabry–Pérot and two-beam interference and are expressed as follows^30^:

1 $$\Delta {{\varnothing }}_{\text{FP}}={{\varnothing }}_{a}+{{\varnothing }}_{c}-\sum_{i={i}_{th}}\frac{4\pi {n}_{i}{d}_{i}cos\theta }{\lambda }=2\pi m$$

2 $$\Delta {\varnothing }_{TI}={\varnothing }_{c}-\frac{4\pi {n}_{{Alq}_{3}}{d}_{{Alq}_{3}}cos\theta }{\lambda }=2\pi m$$

where $\Delta {{\varnothing}}_{\text{FP}}$ and $\Delta {\varnothing}_{TI}$ denote the phase terms of the Fabry–Pérot and two-beam interference, and ${{\varnothing }}_{a}$ and ${{\varnothing }}_{c}$ represent the phase changes that occur with reflections at the anode/organic and cathode/organic interfaces, respectively. Furthermore, ${n}_{i}$ and ${n}_{{Alq}_{3}}$ denote the refractive index of the ${i}_{th}$ layer and Alq_3_, respectively, and ${d}_{i}$ and ${d}_{{Alq}_{3}}$ denote the thickness of the ${i}_{th}$ layer and Alq_3_, respectively. Moreover, λ is the wavelength of the emitted light, and *m* is an integer. The cavity length of the MC-OLEDs is the sum of the *d*_*i*_ values of the layers (upper IZO + organic layers) between the two electrodes. In this work, we deposited a 40-nm-thick IZO layer with a refractive index of 2.0 onto the Ag electrode and used Alq_3_ as the EML with an emission wavelength of 540 nm. The average refractive index of organic materials is 1.75^23^. The cavity length of MC-OLEDs is calculated by substituting the values into Eq. (1). Thus, we obtain a cavity length of 110 nm when *m* = 0 (1st order) and 264 nm when *m* = 1 (2nd order). The cavity length of the 1st order (without considering the thickness of the IZO layer (70 nm)) is excessively thin in that it is less than 100 nm, which would render OLEDs with this internal diffusion layer unstable. Consequently, we fabricated MC-OLEDs with a 2nd-order cavity length. The structure of an MC-OLED additionally needs to take into consideration the two-beam interference within the structure and also necessitates the thickness of the Alq_3_ layer, which constitutes the EML and ETL, to be calculated. This was accomplished by using Eq. (2) to obtain a value of 75 nm. The calculated cavity length was compared with the actual cavity length by analyzing the EL spectra. This required us to fabricate MC-OLEDs with a cavity length that could be adjusted by varying the thickness of the HTL (HATCN + NPB). Ultimately, an EL spectrum with the desired emission peak at 540 nm was obtained for an MC-OLED with a cavity length of 270 nm. (Experimental details appear in Supplementary Fig. S2 and Supplementary Table S2 online).

**Figure 4:**
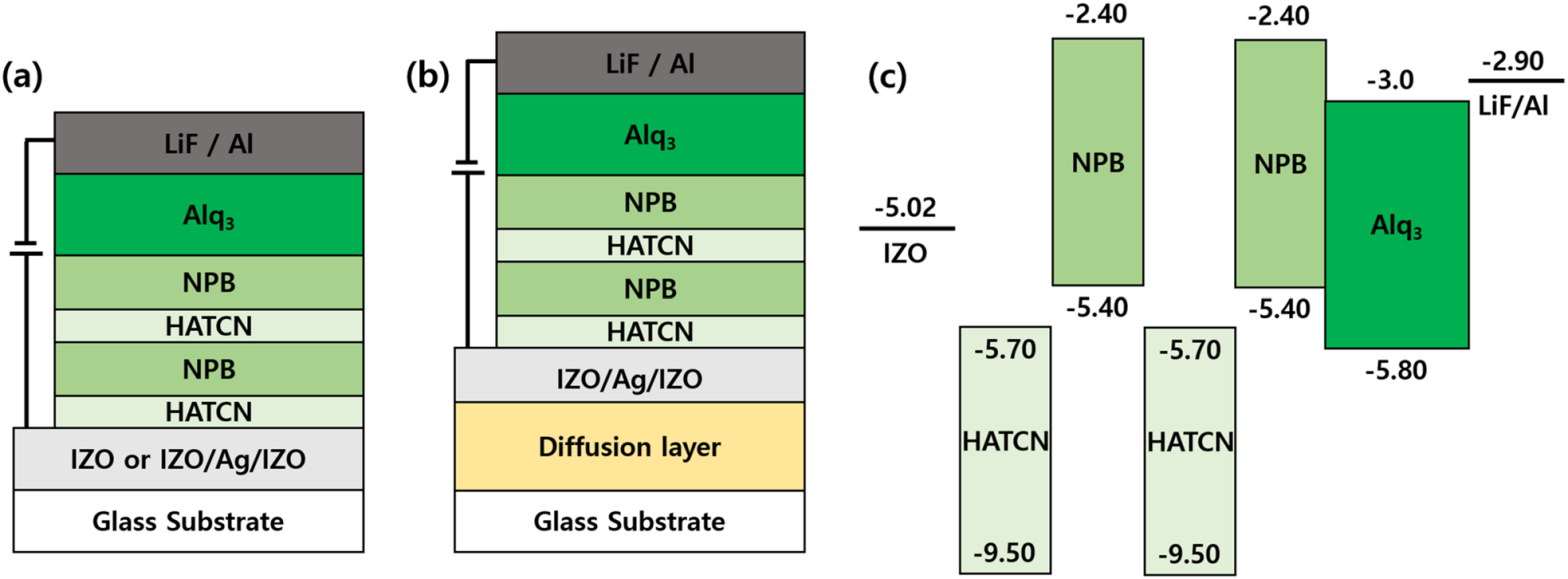
Schematic of the structure of the OLEDs (**a**) without a diffusion layer (both Non-cavity and Micro-cavity devices, and (**b**) MC-OLEDs with a diffusion layer (Device A and B, fabricated under different conditions); (**c**) energy band diagram of materials for OLEDs.

Figure 4 shows the structures of the OLEDs that were fabricated to estimate the effect of the diffusion layer as well as the energy band diagram of the organic materials that constitute the OLEDs. A cavity length of 270 nm and thickness corresponding to 75 nm of Alq_3_ proved to be suitable, based on the cavity length experiment and calculation. Thus, we constructed a 155-nm-thick HTL via repeated deposition of the HATCN/NPB layer because HATCN is a strong *n*-type material. In other words, the HATCN layer extracts electrons from the NPB layer to enable efficient hole transport in the latter layer. Therefore, even a thick HTL can prevent the operating voltage from increasing^31^. Additionally, a Non-Cavity device that includes a transparent IZO anode was fabricated with the same organic materials and thickness to allow us to compare the device characteristics of the non-cavity and micro-cavity structures. The structures of the OLEDs that were fabricated for this study are as follows:

- Non-Cavity device: Glass/IZO (200 nm)/HATCN (20 nm)/NPB (60 nm)/HATCN (15 nm)/NPB (60 nm)/Alq_3_ (75 nm)/LiF (0.5 nm)/Al (100 nm).
- Micro-Cavity device: Glass/IZO (40 nm)/Ag (20 nm)/IZO (40 nm)/HATCN (20 nm)/NPB (60 nm)/HATCN (15 nm)/NPB (60 nm)/Alq_3_ (75 nm)/LiF (0.5 nm)/Al (100 nm).
- Device A (Diffusion A + MC): Glass/1750 rpm diffusion layer/IZO (40 nm)/Ag (20 nm)/IZO (40 nm)/HATCN (20 nm)/NPB (60 nm)/HATCN (15 nm)/NPB (60 nm)/Alq_3_ (75 nm)/LiF (0.5 nm)/Al (100 nm).
- Device B (Diffusion B + MC): Glass/2000 rpm diffusion layer/IZO (40 nm)/Ag (20 nm)/IZO (40 nm)/HATCN (20 nm)/NPB (60 nm)/HATCN (15 nm)/NPB (60 nm)/Alq_3_ (75 nm)/LiF (0.5 nm)/Al (100 nm).

### EL and viewing angle characteristics of MC-OLEDs with a diffusion layer

**Figure 5:**
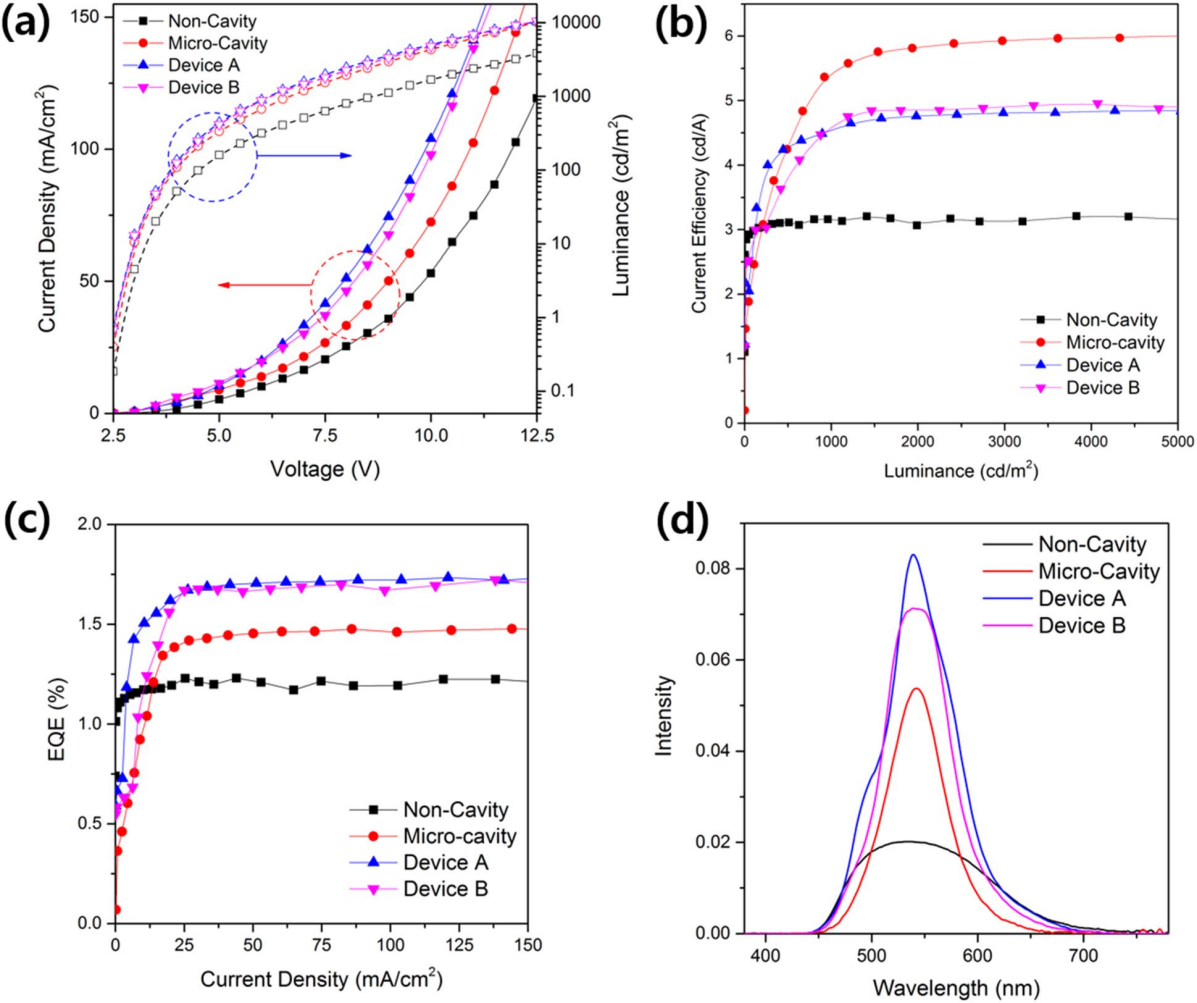
EL characteristics of devices (**a**) current density-voltage-luminance curve, (**b**) current efficiency–luminance curve, (**c**) external quantum efficiency–current density curve, and (**d**) EL spectra of devices.

Figure 5 shows the EL characteristics of the fabricated devices: the current density and luminance as a function of voltage (Fig. 5a), the current density based on the luminance (Fig. 5b), the external quantum efficiency (EQE) based on the current density (Fig. 5c), and the EL intensity as a function of the wavelength (Fig. 5d). As shown in Fig. 5a, MC-OLEDs of which the sheet resistance has been lowered by inserting a Ag metal electrode between the IZO layers exhibit higher current density compared with that of the Non-Cavity device. Additionally, Devices A and B (MC-OLEDs with the additional diffusion layer) do not exhibit a perfectly flat surface despite the insertion of the ZnO semi-planarization layer. A corrugated OLED reportedly has a higher current density than a planar OLED^32^. This phenomenon is also apparent in our work. The current density also increased by increasing the speed at which the diffusion layer was deposited from 1500 to 2500 rpm. (Experimental details are provided in Supplementary Fig. S1 online). Therefore, given the slight corrugation remaining on the surface, the current densities of Device A and B are higher than that of the Micro-Cavity device. Furthermore, the experiments confirmed that the luminance characteristics of the MC-OLEDs with a micro-cavity structure were superior to those of the Non-Cavity device. The variation in the current efficiency as a factor of the luminance is understood as follows: similar to the luminance, the current efficiency of the MC-OLEDs is higher than that of the Non-Cavity device, and the Micro-Cavity device has the highest value among the MC-OLEDs (Non-Cavity: 3.16 cd/A, Micro-Cavity: 5.4 cd/A, Device A: 4.54 cd/A, Device B: 4.57 cd/A at 1000 cd/m^2^). The experiment was designed to measure the current efficiency based on the luminance in the direction normal to the surface. Thus, the current efficiencies of Device A and B, which exhibit scattered light via the diffusion layer, are smaller compared with the efficiency of the Micro-Cavity device. Conversely, as shown in Fig. 5c, recalculation of the EQE by using the angular emission pattern (measured every 10°) reveals a tendency that differs from that of the current efficiency. The tendency whereby MC-OLEDs exhibit higher EQE values compared with those of the Non-Cavity device as a result of micro-cavity resonance is similar to that of the current efficiency. However, in contrast to the tendency exhibited by the current efficiency, the MC-OLEDs (Devices A and B), which include the diffusion layer, have EQE values that are 17.2% and 14.5% higher, respectively, compared with those of the Micro-Cavity device (Non-Cavity: 1.21%, Micro-Cavity: 1.45%, Device A: 1.7%, Device B: 1.66% at 50 mA/cm^2^). These results demonstrate that the light emitted by the MC-OLEDs in the direction normal to the surface is effectively diffused owing to the inserted diffusion layer and that the light extraction efficiency improved such that light is observed at all viewing angles. The reasons for the enhancement in the light efficiency include a decrease in the waveguide mode at the glass/IZO interface because of the insertion of the diffusion layer and a decrease in the SPP mode as a result of the corrugated surface of the interface between the upper reflective electrode and the organic structure^33^. Also, it is well known that the diffusion layer enhances the light extraction efficiency by a corrugated structure. The corrugated structure reduces waveguide mode and SPP mode^34–37^. Franky So et al. reported the 63% of EQE by applied high-refractive-index corrugated substrate^34^. The EL spectra of the fabricated devices (Fig. 5d), show that the MC-OLEDs with a micro-cavity structure exhibit narrower and higher intensity spectra compared with that of the Non-Cavity device (the EL spectra of all devices were measured at 8 V).

**Figure 6:**
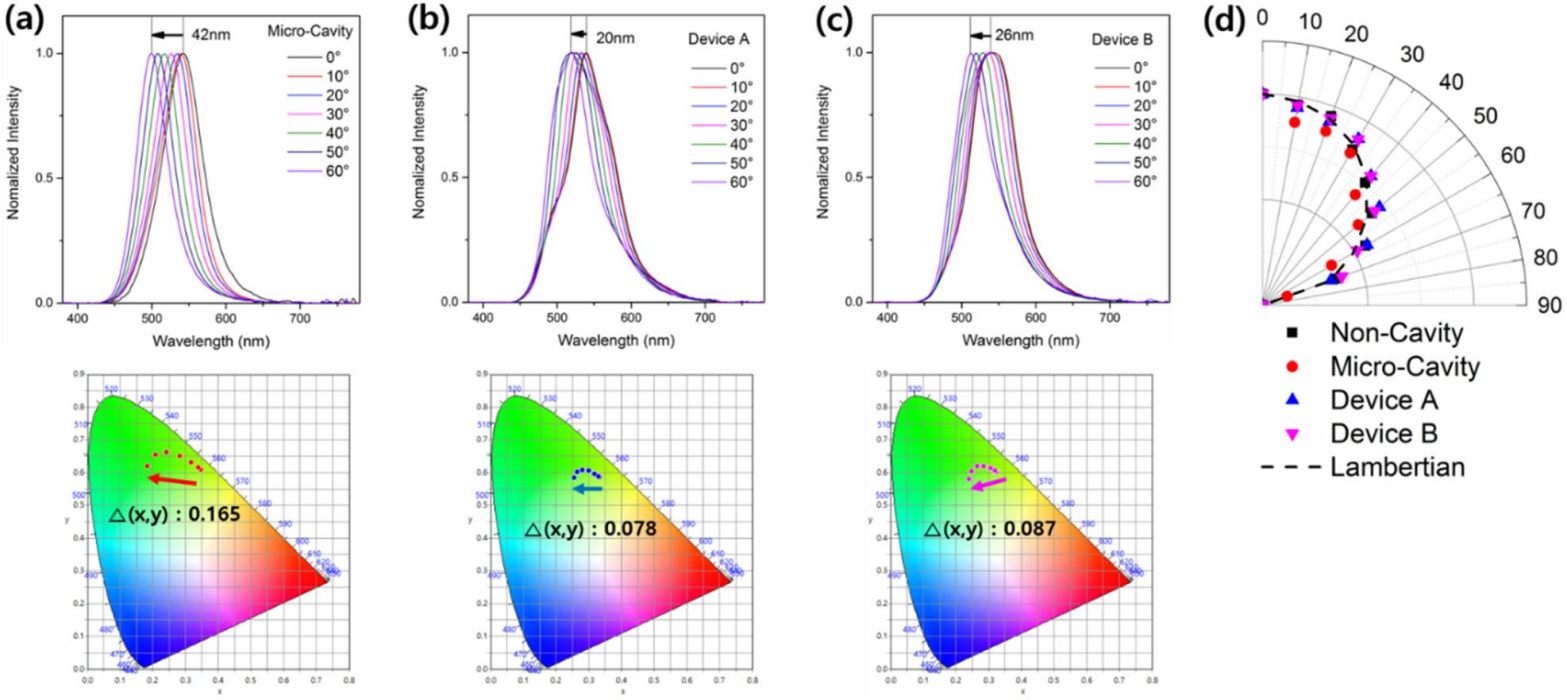
Normalized EL spectra (top) and color coordinates (bottom) showing the shift depending on the viewing angle of the (**a**) Micro-Cavity device, (**b**) Device A, and (**c**) Device B, and (**d**) angular emission pattern of the Non-Cavity device, Micro-Cavity device, Device A, and Device B compared with the Lambertian distribution.

Finally, we analyze the viewing angle characteristics of the fabricated devices. Figure 6a–c show the dependency of the EL spectra (top) and color coordinates (bottom) of the MC-OLEDs on the viewing angle. The strong micro-cavity resonance between the Ag anode and Al cathode when the viewing angle changes from 0° to 60° causes the peak of the Micro-Cavity device to undergo blue shift of 42 nm. However, Device A and B with the diffusion layer exhibit peak shifts of 20 and 26 nm, respectively, this reduced peak shift from 42 nm to 20–26 nm (average − 45%) indicating that the peak shift is reduced by the scattering effect of the diffusion layer. This means that the cavity effect of MC-OLED was reduced in overall viewing angle. Similarly, considering the variation in the color coordinates based on the viewing angle, Device A and B (with the diffusion layer) exhibit less color change compared with the Micro-Cavity device (Micro-Cavity: 0.165, Device A: 0.078, Device B: 0.087). Figure 6d shows the angular emission pattern of the fabricated devices. The Non-Cavity device exhibits an emission pattern similar to the Lambertian distribution, whereas that of the Micro-Cavity device is narrower compared with the Lambertian distribution. Conversely, Device A and B (with the diffusion layer) exhibit an emission pattern that is similar to that of the Lambertian distribution or wider than that of the Micro-Cavity device because of the effect of the diffusion layer as mentioned above. The EL and viewing angle characteristics of the fabricated devices are summarized in Table 3. The diffusion characteristics of the corrugation structure showed a trade-off characteristic between the light extraction and micro-cavity effect. The full-width-half-maximum (FWHM), which indicates the degree of micro-cavity effect, was 60 nm in MC-OLED and increased to 71 nm in Devices A and B. Also, in Fig. 5, Devices A and B show a broad emission spectrum by increasing light extraction with the corrugated diffusion layer. Finally, Devices A and B show the improved EL efficiency and viewing angle characteristics by improved light extraction while those have slightly decreased color purity (broaden emission spectrum, increased FWHM) by decreased cavity effect.

**Table 3:** Summary of device characteristics.

	Non-Cavity	Micro-Cavity	Device A	Device B
Turn on voltage (at 1 cd/m^2^)	2.78 V	2.65 V	2.56 V	2.56 V
Current efficiency (at 1000 cd/m^2^)	3.16 cd/A	5.4 cd/A	4.54 cd/A	4.57 cd/A
EQE (at 50 mA/cm^2^)	1.21%	1.45%	1.7% (+ 17.2%)^a^	1.66% (+ 14.5%)^a^
Full width half maximum (FWHM)	147 nm	60 nm	71 nm	71 nm
Δ(x,y) (0° → 60°)	0.057	0.165	0.078	0.087
EL spectra peak shift (0° → 60°)	8 nm	42 nm	20 nm	26 nm

^a^Efficiency enhancement ratio when compared to that of the Micro-Cavity device.

## Conclusion

We inserted a diffusion layer into an MC-OLED with a micro-cavity structure to improve the viewing angle characteristics and light extraction efficiency of the device. The diffusion layer is composed of randomly distributed PMMA (a transparent polymer) nanostructures (formed by RIE) and a ZnO semi-planarization layer with a high refractive index. The height and density of the fabricated structure were controlled by regulating the thickness of the PMMA deposition (by varying the spin-coating speed). The ZnO semi-planarization layer was deposited on the PMMA nanostructures by using RF sputtering. The insertion of the fabricated diffusion layer into the MC-OLEDs increased the EQE by as much as 17% compared with the device without the diffusion layer (the Micro-Cavity device) because the waveguide and SPP modes decreased. Additionally, the viewing angle characteristics of the devices with the diffusion layer improved relative to those of the Micro-Cavity device. Furthermore, the dependency of the color coordinates and maximum intensity of the EL peaks on the viewing angle decreased, and the angular emission pattern widened such that it more closely approximated the Lambertian distribution.

## Methods

### Characterization and measurement

The surface morphologies of the diffusion layer with the nano-sized structures were examined by using field emission scanning electron microscopy (FE-SEM, S-4800, Hitachi High-Technologies, Inc.). Optical transmittance was measured with a UV–Vis spectrometer (Cary-5000, Agilent Technologies, Inc.), and the EL characteristics of the OLED devices were measured by using a spectroradiometer (Spectra Scan PR-670, Photo Research, Inc.) in a dark box with a source meter (Model 237, Keithley Instruments, Inc.) without any encapsulation.

### Fabrication of the diffusion layer with the nano-sized structures

**Figure 7:**
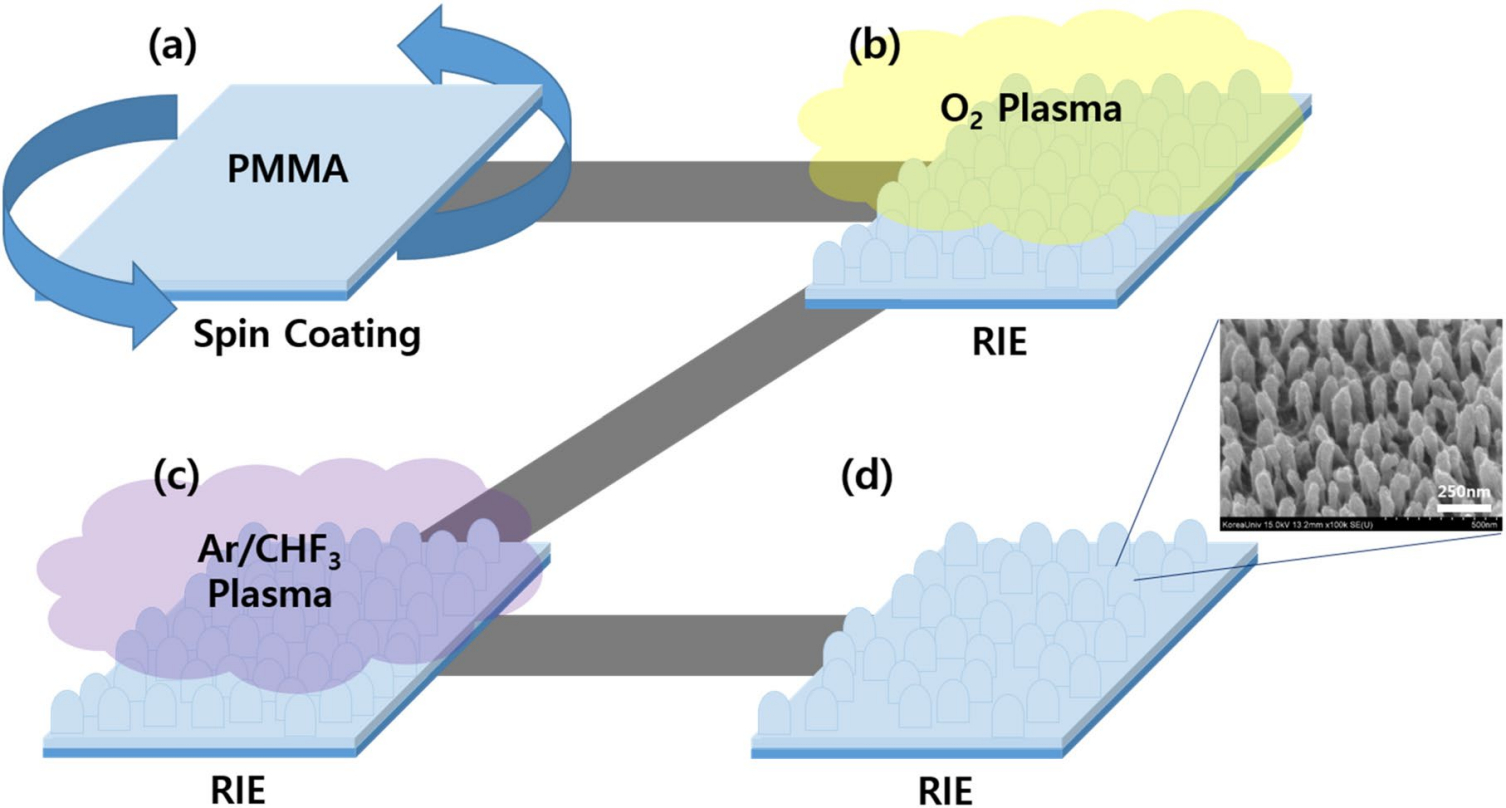
Fabrication of the diffusion layer with the nano-sized structures: (**a**) PMMA is spin coated onto the substrate, (**b**) PMMA etching via RIE O_2_ plasma, (**c**) PMMA etching via RIE Ar/CHF_3_ plasma, and (**d**) fabricated diffusion layer with the nano-sized structures.

Figure 7 shows the process whereby the diffusion layer with the nano-sized structures was fabricated by using poly(methyl methacrylate) (PMMA, Microchem Co.) (a transparent polymer) and an SEM image of the fabricated nano-sized structures. The detailed steps are as follows. First, 0.5 T thick Eagle XG glass substrates (Corning, Inc.) were immersed in acetone, methanol, and deionized water and subsequently cleaned in an ultrasonic bath for 15 min. Then, the substrates were dried in a stream of nitrogen to remove residual solution on the surface and dried in an oven at 110 °C. Subsequently, PMMA was dropped onto each glass substrate with a micropipette and a 600-nm-thick layer was formed on the substrate using spin coating. The glass substrate coated with PMMA was heated at 170 °C for 40 min on a hotplate to cure the PMMA (Fig. 7a), and dry etching (RIE) was subsequently carried out with plasma energy to form the diffusion layer with the nano-sized structures. The plasma gas used for dry etching was (in sequence) O_2_ and a gas mixture consisting of Ar/fluoroform (CHF_3_) in a ratio of 4: 1 to form the columnar nano-sized structures (Fig. 7b,c)^38,39^. The inset in Fig. 7d shows a FE-SEM image of the shape of the PMMA nano-sized structures fabricated via dry etching. Following the fabrication of the diffusion layer, RF sputtering was employed to deposit a semi-planarization layer consisting of a material with a high refractive index (ZnO) with a thickness of 400 nm to prevent an electrical short circuit because of the surface roughness resulting from the nano-sized structures in the diffusion layer.

### Materials

Specifically, IZO and Ag were used as the anode, 1,4,5,8,9,11-hexaazatriphenylene-hexacarbonitrile (HATCN) and N,N′-bis(naphthalen-1-yl)-N,N′-bis(phenyl) benzidine (NPB) as a hole transport layer (HTL), tris-(8-hydroxyquinoline)aluminum (Alq_3_) as the emitting layer (EML) and electron transport layer (ETL), lithium fluoride (LiF) as the electron injection layer, and aluminum (Al) as the cathode. All the organic materials were sublimed level (> 99.5%) and purchased from Luminescence Technology Corp., and the inorganic compounds and metals were purchased from Kojundo Chemical Laboratory Co., Ltd.

### Fabrication of OLEDs

Conventional and micro-cavity OLEDs were fabricated to investigate the bottom emission properties. The conventional OLED had an IZO (200 nm) anode whereas the MC-OLEDs had IZO/Ag/IZO (40 nm/20 nm/40 nm) anodes. These two devices had the same organic layers and cathode structure, namely HATCN (20 nm)/NPB (60 nm)/HATCN (15 nm)/NPB (60 nm)/Alq_3_ (75 nm)/LiF (0.5 nm)/Al (100 nm). The IZO was deposited via RF sputtering, and the organic materials and metal were deposited by thermal evaporation under high vacuum (approximately 3 × 10^–6^ Torr). The deposition rates of all the organic materials and metals corresponded to 1 and 3 Å/s, respectively.

## Supplementary Information

Supplementary Information.
