# Supplementary material for: Enhanced light extraction efficiency and viewing angle characteristics of microcavity OLEDs by using a diffusion layer
Source: Sci Rep. 2021 Feb 9;11:3430. doi: 10.1038/s41598-021-82753-9 (PMC7873264; doi:10.1038/s41598-021-82753-9)
Supplement: Supplementary file 1 — Supplementary Information. [file 41598_2021_82753_MOESM1_ESM.docx]

Enhanced Light Extraction Efficiency and Viewing Angle Characteristics of Microcavity OLEDs by Using a Diffusion Layer

(Supplementary Information)

Cheol Hwee Park^a,‡^, Shin Woo Kang^a,b,‡^, Sun-Gyu Jung^a^, Dong Jun Lee^a^, Young Wook Park^b,*^, and Byeong-Kwon Ju^a,*^

^a^ Display and Nanosystem Laboratory, Department of Electrical Engineering, Korea University, 145, Anam-ro, Seongbuk-gu, Seoul 02841, Republic of Korea

^b^ Nano and Organic-Electronics Laboratory, Department of Display and Semiconductor Engineering, Sun Moon University, Asan, Chungcheongnam-do 31460, Republic of Korea

^*^Corresponding authors: E-mail: zerook@sunmoon.ac.kr (Y.W. Park), bkju@korea.ac.kr (B.-K. Ju)

^a^ Phone No.: +82-2-3290-3237,

^a^ Fax. No.: +82-2-3290-3791

‡These authors contributed equally to this work

**1. Light extraction effect of diffusion layer in conventional OLEDs**


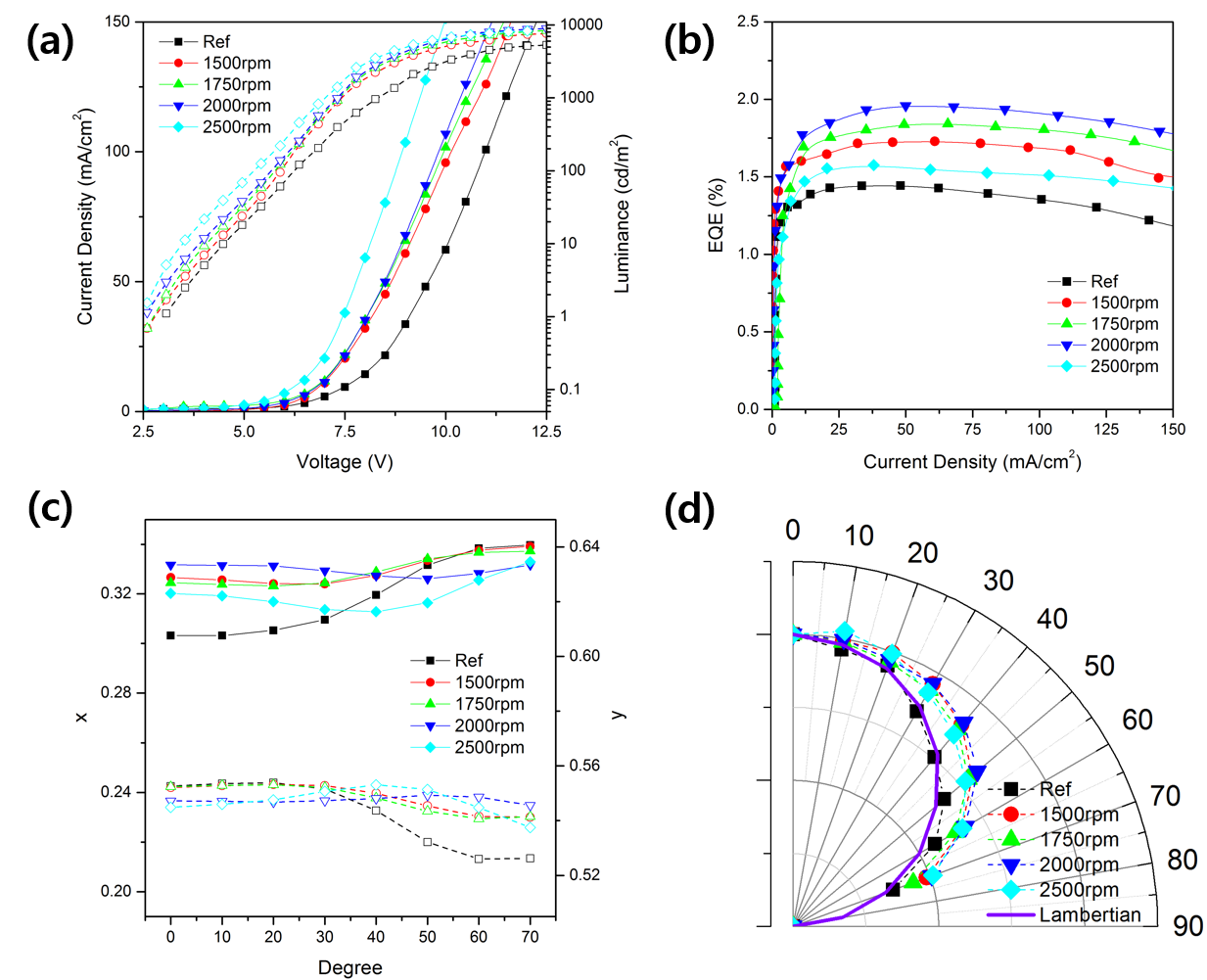


**Figure S1.** (a) current density-voltage-luminance curve, (b) external quantum efficiency-current density curve of OLEDs with diffusion layer, (c) color coordinate of OLEDs according to viewing angle and (d) angular emission pattern of the OLEDs with diffusion layer, to compare with the Ref device and Lambertian distribution.

We confirmed the scattering effect of the structure by inserting nano-sized PMMA structures into OLED devices using IZO transparent electrodes. The structure of the fabricated devices are as follows:

**Ref**: Glass/IZO (200 nm)/NPB (60 nm)/Alq_3_ (80 nm)/LiF (0.5 nm)/Al (100 nm)

**X rpm device**: Glass/diffusion layer (X rpm)/IZO (200 nm)/NPB (60 nm)/Alq_3_ (80 nm)/LiF (0.5 nm)/Al (100 nm)

The diffusion layer is composed of an etched PMMA structure fabricated using a coating speed of X rpm and a 400 nm ZnO semi-planarization layer. The EL and viewing angle characteristics of the fabricated devices are presented in Fig. S1 and Table S1.

As shown in Fig. S1 (a), the devices with a diffusion layer have a corrugated structure owing to the surface roughness of the diffusion layer and have a relatively higher current density than the Ref device. The tendency of the luminance characteristic is similar to the current density characteristic. The curve of the EQE as a factor of the current density confirms that the OLEDs with the inserted diffusion layer have higher efficiency than the Ref device. Of these, the device with the diffusion layer fabricated at 2000 rpm had the highest efficiency, and the 1750 rpm device had the second highest efficiency. The reason why the devices fabricated at 1500 rpm and 2500 rpm are less efficient than the previous two devices is that the density of the PMMA structure is too high for the 1500 rpm device, resulting in an increase in the waveguide mode between the semi-planarization layer and the PMMA structure. In the case of the 2500 rpm device, this is because the height of the formed structure was relatively low, such that the scattering effect was smaller than that of other devices.

Compared to the Ref device, which had a color coordinate shift of 0.0441 when the viewing angle changed from 0 to 60 degrees, the devices with a diffusion layer exhibited very little color coordinate shift according to the viewing angle (1500 rpm: 0.0153, 1750 rpm: 0.0169, 2000 rpm: 0.0037, 2500 rpm: 0.0053). Unlike the Ref device, which has a pattern similar to the Lambertian distribution in the angular emission pattern according to the viewing angle, the emission pattern of the devices with the diffusion layer is broader than the Lambertian because of the scattering effect of the diffusion layer.

In this experiment, the diffusion layers with the PMMA structure were inserted into the device. As a result, the efficiency of the devices fabricated at 1750 rpm and 2000 rpm were improved to the greatest extent with respect to the Ref device. Therefore, these two conditions to fabricate the diffusion layer were used in the main experiments in the manuscript.

**Table S1.** Summary of characteristics of OLEDs with diffusion layer

|  | **Ref** | **1500 rpm** | **1750 rpm** | **2000 rpm** | **2500 rpm** |
| --- | --- | --- | --- | --- | --- |
| **Turn on Voltage (at 1cd/m^2^)** | 3V | 2.65V | 2.65V | 2.4V | 2.3V |
| **EQE (at 50mA/cm^2^)** | 1.44% | 1.72% (+19.4%) | 1.83% (+27.1%) | 1.96% (36.1%) | 1.56% (+8.3%) |
| **Δ(x, y) (0° → 60°)** | 0.0441 | 0.0153 | 0.0169 | 0.0037 | 0.0053 |

**2. Optimization of cavity length of micro-cavity OLEDs (MC-OLEDs)**

To determine the optimal cavity length of the MC-OLED to be fabricated, we analyzed the characteristics of the MC-OLED according to the cavity length by varying the thickness of the HTL. The structure of the fabricated devices are as follows:

**Ref**: IZO (200 nm)/HATCN (30 nm)/NPB (140 nm)/Alq_3_ (75 nm)/LiF (0.5 nm)/Al (100 nm)

**Cavity 1**: IZO (40 nm)/Ag (20 nm)/IZO (40 nm)/HATCN (15 nm)/NPB (140 nm)/Alq_3_ (75 nm)/LiF (0.5 nm)/Al (100 nm) – total cavity length (270 nm)

**Cavity 2**: IZO (40 nm)/Ag (20 nm)/IZO (40 nm)/HATCN (30 nm)/NPB (140 nm)/Alq_3_ (75 nm)/LiF (0.5 nm)/Al (100 nm) – total cavity length (285 nm)

**Cavity 3**: IZO (40 nm)/Ag (20 nm)/IZO (40 nm)/HATCN (40 nm)/NPB (140 nm)/Alq_3_ (75 nm)/LiF (0.5 nm)/Al (100 nm) – total cavity length (290 nm)

As shown in Fig. S2, the devices with a cavity structure have a narrower FWHM than the Ref devices. Among these devices with the cavity, we confirmed that the peak on the EL spectrum of the Cavity 1 device is at 542 nm, which is similar to the emission peak of Alq_3_ used for the emitting layer. The spectral peaks of the Cavity 2 and 3 devices, of which the cavity lengths are longer than those of the Cavity 1 device, underwent red shifts and their peaks occur at 568 nm and 570 nm, respectively. Therefore, MC-OLEDs with a cavity length of 270 nm were fabricated and were used to conduct subsequent experiments.

**
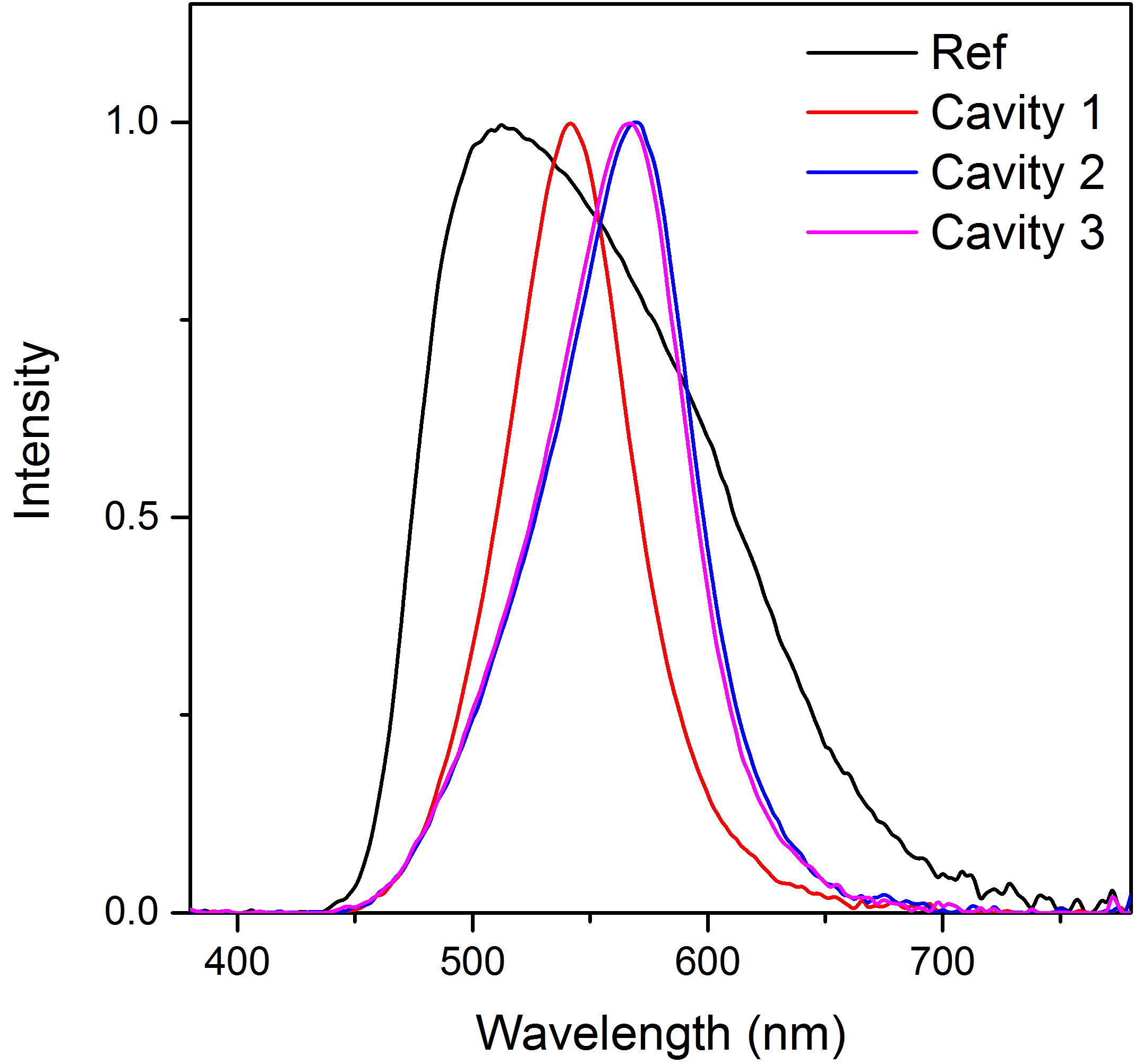
**

**Figure S2.** Normalized EL spectra of MC-OLEDs with various cavity lengths.

**Table S2.** Summary of characteristics of MC-OLEDs EL spectra

|  | **Ref** | **Cavity 1** | **Cavity 2** | **Cavity 3** |
| --- | --- | --- | --- | --- |
| **Cavity length** | 225 nm | 270 nm | 285 nm | 295 nm |
| **Peak wavelength** | 512 nm | 542 nm | 568 nm | 570 nm |
| **FWHM** | 148 nm | 61 nm | 71 nm | 70 nm |
